# Transcatheter edge-to-edge repair of both atrioventricular valves in congenitally corrected transposition of the great arteries: a case report

**DOI:** 10.1093/ehjcr/ytae348

**Published:** 2024-07-16

**Authors:** Alexandru Patrascu, Donat Binder, Peter Schnabel, Kai Weinmann, Ilka Ott

**Affiliations:** Department of Cardiology, Helios Hospital Pforzheim, Kanzlerstrasse 2-6, 75175 Pforzheim, Germany; Private University in the Principality of Liechtenstein (UFL), Triesen, Principality of Liechtenstein; Department of Cardiology, Helios Hospital Pforzheim, Kanzlerstrasse 2-6, 75175 Pforzheim, Germany; Department of Cardiology, Helios Hospital Pforzheim, Kanzlerstrasse 2-6, 75175 Pforzheim, Germany; Department of Cardiology, Helios Hospital Pforzheim, Kanzlerstrasse 2-6, 75175 Pforzheim, Germany; Department of Cardiology, Helios Hospital Pforzheim, Kanzlerstrasse 2-6, 75175 Pforzheim, Germany

**Keywords:** Tricuspid valve, Mitral valve, Transcatheter edge-to-edge repair, Congenitally corrected transposition of the great arteries, Case report

## Abstract

**Background:**

Transcatheter edge-to-edge repair (TEER) for the systemic atrioventricular valve has been anecdotally reported as a viable treatment option in symptomatic inoperable adult patients born with congenitally corrected transposition of the great arteries (ccTGA). However, to date, case reports on TEER treatment of both atrioventricular valves are lacking, especially when considering the present availability of specific mitral and tricuspid valve TEER devices.

**Case summary:**

We present the case of an 84-year-old man with recurrent admissions for acute heart failure due to high-grade regurgitation of both atrioventricular valves. The patient was first diagnosed with ccTGA at this advanced age and underwent a thorough multimodality imaging approach, including transthoracic and transoesophageal echocardiography, cardiac magnetic resonance imaging, cardiac computed tomography, and ventriculography of the systemic ventricle. Due to the high symptom burden despite optimal medical therapy and high doses of diuretics, the heart team recommended TEER, first for the systemic tricuspid valve and later on for the non-systemic mitral valve. Both complex procedures were uneventful and led to considerable improvement in quality of life.

**Discussion:**

Congenitally corrected transposition of the great arteries mostly manifests itself in adulthood and affects both ventricles and atrioventricular valves. In case of anatomical doubts on transthoracic echocardiography, a thorough multimodality imaging work-up is recommended. Transcatheter treatment of both atrioventricular valves seems to be a safe and effective therapeutic option in these often inoperable patients.

**Learning points**

- Multimodality imaging is crucial in the diagnosis of congenitally corrected transposition of the great arteries (ccTGA) and its associated anomalies.
- Conventional heart failure (HF) therapy may not be sufficient for heart failure with reduced ejection fraction in the context of ccTGA, whereas surgical risk is often prohibitive for these patients. Transcatheter intervention of atrioventricular valves may, thus, provide a doable—and often the only—therapeutic resort that can improve functional status in this HF subpopulation.

## Introduction

Congenital heart defects are sometimes not limited to only children and can be discovered at advanced age.

Congenitally corrected transposition of the great arteries (ccTGA) sometimes does not manifest itself until adulthood and affects both ventricles and atrioventricular (AV) valves.^1^ Due to the rarity of this disease, in the context of general adult cardiology, a thorough multimodality imaging work-up seems reasonable. Furthermore, ccTGA patients are often discovered at advanced ages or with signs of advanced systemic ventricle failure and are most frequently deemed inoperable.^2^ In this setting, transcatheter treatment of the systemic tricuspid valve (TV) by transcatheter edge-to-edge repair (TEER) is gaining ground.^3^ However, to date, reports on TEER treatment of both AV valves are lacking. The following case report describes such a unique therapeutic approach, where interventional repair of both AV valves was key in symptom control and avoiding further hospitalizations for acute heart failure, in a highly symptomatic octogenarian diagnosed with ccTGA.

## Summary figure

**Table:** 

Date	Events
2019–21	Several hospitalizations for acute heart failure in a peripheral hospital
	Optimal medical therapy for HFrEF
	Continuous progression of regurgitation of the supposed mitral valve (according to referral letter)
January 2022	Transfer to our department for TEER of the supposed mitral valve
	Signs of acute heart failure, massive oedema, NYHA IV dyspnoea
	Diagnosis of ccTGA
	Heart team discussion
February 2022	TEER of the systemic TV
	Reduction of severe regurgitation to mild
April 2022	Improved NYHA functional class, residual peripheral oedema
	Confirmation of good result after systemic TEER with mild residual regurgitation
	Unchanged severe regurgitation of the non-systemic AV valve
May 2022	TEER of the non-systemic mitral valve
	Reduction of severe regurgitation to mild to moderate
June 2022	30-day follow-up after last TEER
	Mild regurgitation of systemic AV valve, mild-to-moderate regurgitation of the non-systemic valve
	Improvement in QoL, NYHA II, no more peripheral oedema
December 2023	18-month follow-up after both TEER procedures
	No echocardiographic change
	No change in QoL

HFrEF, heart failure with reduced ejection fraction; TEER, transcatheter edge-to-edge repair; NYHA, New York Heart Association; ccTGA, congenitally corrected transposition of the great arteries; QoL, quality of life.

## Case presentation

An 84-year-old Caucasian male was transferred to our institution for TEER of the mitral valve (MV). The patient had a history of recurrent admissions for acute heart failure in a peripheral hospital, where severe functional mitral regurgitation was diagnosed. Supposedly, functional regurgitation was of ventricular cause, as left ventricular (LV) ejection fraction was impaired, according to the medical letter. Upon transfer, the patient was stable, but complained of shortness of breath on minimal activity and intermittently at rest. Physical examination showed massive peripheral oedema resistant to diuretics and some inspiratory crackles. Optimal medical therapy, in dosages tolerated by the patient, was started several months prior and included dapagliflozin 10 mg daily, sacubitril/valsartan 24/26 mg twice daily, metoprolol 47.5 mg daily, and spironolactone 25 mg daily. On top, intravenous furosemide dose was increased to 80 mg twice daily at transfer. The patient’s medical history was significant for type 2 diabetes, chronic kidney failure stage 3A, permanent atrial fibrillation in need of oral anticoagulation with 5 mg apixaban twice daily, gout, and benign prostatic hyperplasia.

**Figure 1:**
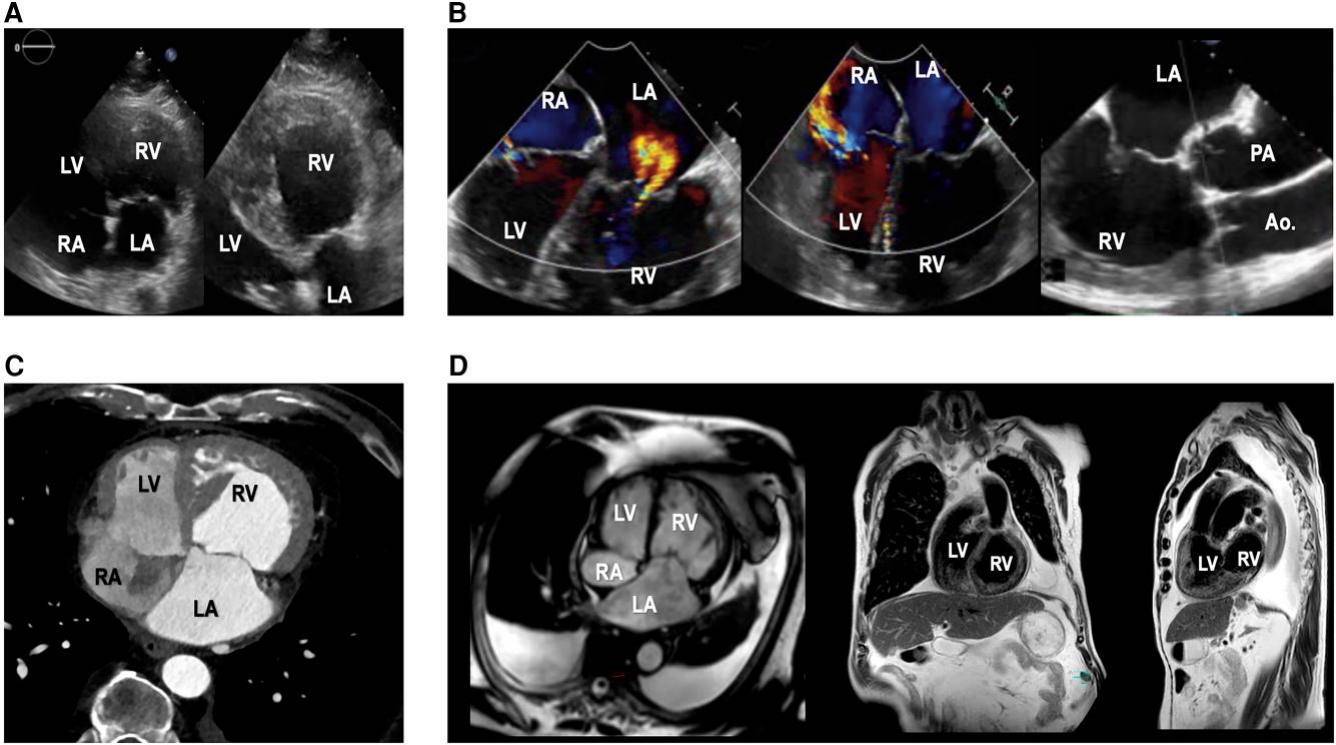
Morphological features in a case of congenitally corrected transposition of the great arteries. Transthoracic (*A*), transoesophageal (*B*), cardiac computed tomography (*C*), and cardiac magnetic resonance imaging (*D*) multimodality imaging in an 84-year-old congenitally corrected transposition of the great arteries patient. Notice the apical displacement of the left-sided tricuspid valve, the parallel course of the great vessels, the morphology of the systemic right ventricle, and the significant regurgitation of both atrioventricular valves. RA, right atrium; RV, right ventricle; LA, left atrium; LV, left ventricle; Ao, aortic artery; PA, pulmonary artery.

Transthoracic echocardiography (TTE) was performed at admission and showed abnormal anatomy (*Figure 1A*; Supplementary material online, *Video S1*), most notably unusual depth between AV annular plane and cardiac apex, in side-by-side comparison. Also, both AV valves had significant regurgitation, the left-sided subaortic ventricle demonstrated increased trabeculation, and the right-sided subpulmonary ventricle showed hypertrophy of the free wall. Incorrect echo probe positioning and a sternal scar were ruled out. The following day, transoesophageal echocardiography (TEE) confirmed moderate-to-severe functional regurgitation of the left-sided AV valve, under high dose sedation, while the right-sided valve suffered from severe eccentric regurgitation due to leaflet prolapse (*Figure 1B*; Supplementary material online, *Video S2*). Also, the ascending aorta and pulmonary artery showed a parallel course. Using cardiac computed tomography (CT; *Figure 1C*; Supplementary material online, *Video S3*) and magnetic resonance imaging (MRI; *Figure 1D*), we were able to establish normal situs solitus with levocardia and rule out other anomalies including intracardiac shunts, valvular stenoses, and bilateral outflow tract obstruction. Therefore, the initial suspicion of ccTGA was confirmed. Typical features include the following: apical displacement of the left-sided AV valve, parallel course of the great arteries, and a favourable double discordance, both AV and ventriculoarterial. The valve that the patient was referred for was not the MV, but in fact the systemic TV, attached to both the systemic subaortic right ventricle (RV) and the left atrium. The ‘true’ MV was the right-sided non-systemic AV valve, connected to the subpulmonary LV and the right atrium, and showed severe primary regurgitation due to anterior leaflet (AML) prolapse. A ventriculography of the subaortic ventricle as part of invasive diagnostics confirmed a severe systolic impairment (see Supplementary material online, *Video S4*). Also, an abnormal origin of the circumflex artery from the right coronary artery was present, with a small fistula flowing towards the posterior wall of the pulmonary artery (see Supplementary material online, *Video S5*). Of note, on cardiac MRI, biventricular ejection fraction was 25% (subaortic RV, absolute end-diastolic volume 214 mL, indexed end-diastolic volume 123 mL/m^2^) and 65% (subpulmonary LV, absolute end-diastolic volume 116 mL, indexed end-diastolic volume 66 mL/m^2^), respectively. Also, the subaortic ventricle showed typical RV morphological features, like the presence of trabecula septomarginalis, and attachment of the TV septal leaflet to the interventricular septum. Hypertrophy of the subaortic RV was caused by long-term failure to adapt to the demands of the systemic circulation, while hypertrophy of the subpulmonary LV was attributed to pulmonary hypertension (50 mmHg systolic pulmonary artery pressure on TTE).

**Figure 2:**
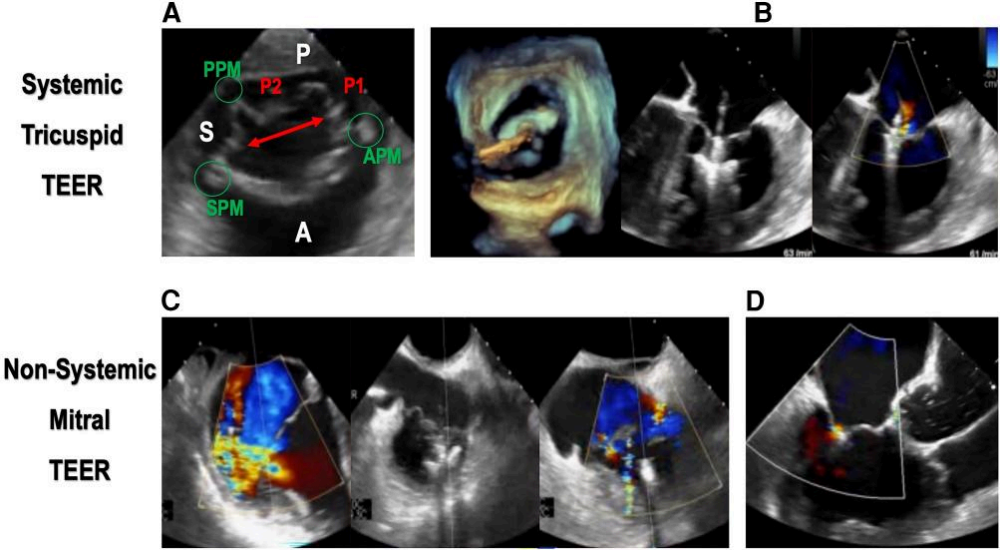
Transcatheter edge-to-edge repair of both atrioventricular valves in the same congenitally corrected transposition of the great arteries patient. Systemic tricuspid transcatheter edge-to-edge repair by central implantation of one MitraClip XTW™ (*A* and *B*) between the anterior scallop (P1) of the posterior leaflet (P) and the septal leaflet (S, red arrow). Non-systemic mitral transcatheter edge-to-edge repair by implantation of TriClip XTW™ with regurgitation reduction to mild to moderate (*C*). Follow-up shows excellent systemic transcatheter edge-to-edge repair result at 18 months (*D*). A, anterior leaflet; SPM/APM/PPM, right ventricular septal/anterior/posterior papillary muscles.

The case was discussed by the heart team. The patient was considered inoperable, as timing of corrective surgery^4^ was off by seven to eight decades, and the surgical risk was deemed prohibitive (EuroSCORE II 8.5%, frailty). Therefore, percutaneous treatment of the systemic TV by TEER was suggested. For this, a MV-specific device (MitraClip™ XTW, Abbott Medical) was chosen, as more angulation of the steerable guide catheter was needed to reach the apically displaced systemic AV valve (see Supplementary material online, *Video S6*). Other particularities of this TV were its ‘four-leaflet configuration’, with two posterior scallops, and the presence of the septal leaflet on the mirrored side of the transgastric ‘en face’ view (*Figure 2A*), which further increased procedural complexity. Finally, with the help of 3D TEE guidance, the device was implanted centrally, along the posteroseptal commissure, by grasping both the septal leaflet and the more anteriorly positioned scallop of the posterior leaflet. This resulted in bicuspidalization of the TV (see Supplementary material online, *Video S7*) and reduction of regurgitation from severe to mild (*Figure 2B*; Supplementary material online, *Video S8*). The mean pressure gradient by the end of the 150-min procedure was 2 mmHg, with 100-min device time and 4137 cGy total radiation dose. No complications occurred, and the patient was discharged 2 days later.

After 2 months, he reported significant improvement in shortness of breath, from initial New York Heart Association (NYHA) functional classes III and IV at transfer to class II at follow-up. However, he complained of residual peripheral oedema, despite high dosages of diuretics. Echocardiography confirmed both the excellent result after interventional systemic TEER and the remaining severe regurgitation of the non-systemic MV. The heart team recommended again percutaneous treatment by TEER. This time, a TriClip™ XTW device (Abbott Medical) was employed. Due to the primary nature of the regurgitation, caused by AML prolapse, clip implantation was very challenging, both from an interventional and imaging perspective. First, the usually thicker MV leaflets, always well visible on 3D TEE images, appeared thinner than expected, which speaks to the adaptations to the low-pressure environment of the non-systemic circulation. Second, the highly elongated AML showed above than average billowing area and prolapsing depth, which led to the device getting stuck in the chordae in the beginning. Finally, the clip was successfully placed in a central position with a slight lateral tilt, owing to the asymmetrical lengths of both leaflets (*Figure 2C*; Supplementary material online, *Video S9*). This resulted in regurgitation reduction from severe to mild to moderate. Procedural data included a total time of 112 min, device time of 55 min, radiation dose of 2093 cGy, and postprocedural MV gradient of 1.5 mmHg.

Currently, 18 months after the second edge-to-edge procedure, the patient has considerably less dyspnoea (NYHA I and II), with only trace peripheral oedema. Echocardiographic results, including biventricular ejection fraction, remain unchanged, with mild residual TV regurgitation and mild-to-moderate MV regurgitation (*Figure 2D*; Supplementary material online, *Video S10*). Of note, the iatrogenic atrial septal defect, caused by transseptal puncture during the first TEER procedure, remains haemodynamically non-significant, as the pulmonary to systemic blood flow ratio (Qp/Qs) measures 1.3 on TTE. Initial heart failure medication was continued, with no further need for diuretics at present.

## Discussion

This case report highlights the need for an extensive multimodality imaging approach in patients with suspicion of ccTGA and underscores the value of transcatheter interventions for AV valves in this small cohort of heart failure with reduced ejection fraction (HFrEF) patients.

In terms of imaging, while, *in utero*, foetal ultrasound can diagnose ccTGA,^5^ in adults, extensive multimodality imaging using TEE, cardiac MRI, and sometimes CT, on top of TTE, greatly enhances morphological and functional information and can detect associated anomalies.^6^ This is especially important, as the natural history and clinical presentation of ccTGA patients is determined by associated malformations.^1,2^ Even in the case of isolated ccTGA, only around 50% of patients reach the age of 60.^1,7^ Current guidelines^7,8^ recommend typical HFrEF medical therapy and palliative TV replacement in selected cases, as corrective surgery, e.g. ‘double switch’, is considered futile with increasing age. However, as this case report shows, conventional anti-failure treatment may not be adequate in case of HFrEF and congenital heart disease, and thus, transcatheter procedures, such as TEER of AV valves, may provide the best available option in such inoperable patients. Nonetheless, despite the exponential increase in TEER procedures for both AV valves in subjects with normal anatomy, to date, there is only anecdotal experience in ccTGA patients.^9^ Recently, the first published case series on TEER of the systemic AV valve^3^ demonstrated feasibility, with five out of six patients showing great long-term results. Therefore, TEER appears an effective and safe option in these usually inoperable patients. As for the non-systemic MV, no published case report exists so far.

In conclusion, ccTGA mostly manifests itself in adulthood and affects both ventricles and AV valves. We believe a thorough cardiac imaging work-up is needed in order to understand morphology and possible associated malformations. This is the first case report on TEER of both AV valves, which appears to be a valuable therapeutic option in ccTGA.

## Supplementary Material

ytae348_Supplementary_Data

## Data Availability

Data available on request: the data underlying this article will be shared on reasonable request to the corresponding author.

## Lead author biography

Alexandru Patrascu is a young interventional cardiologist working as an attending doctor at the Helios Hospital in Pforzheim, Germany. After completing his training in general cardiology in Robert Bosch Hospital, Stuttgart, Germany, he was awarded the EAPCI Grant and started his interventional career in Georges Pompidou, Paris, France. After a second fellowship in interventional and structural cardiology at the Andreas Gruentzig Heart Cath Laboratories, Zurich, Switzerland, he joined the interventional team of the academic hospital in Pforzheim. His research interests include percutaneous treatment in structural heart disease, as well as multimodality imaging in valvulopathies.

## Notes

**Consent:** The authors confirm that written consent for submission and publication of this case report, including images and associated text, has been obtained from the patient in line with COPE guidance.

**Funding:** None declared.
